# Anti-tumor activity of the beta-adrenergic receptor antagonist propranolol in neuroblastoma

**DOI:** 10.18632/oncotarget.1083

**Published:** 2013-11-04

**Authors:** Jennifer K Wolter, Nikolaus E Wolter, Alvaro Blanch, Teresa Partridge, Lynn Cheng, Daniel A. Morgenstern, Monika Podkowa, David R. Kaplan, Meredith S. Irwin

**Affiliations:** ^1^ Departments of Pediatrics and Medical Biophysics, University of Toronto; ^2^ Cell Biology Program, Hospital for Sick Children; ^3^ Department of Otolaryngology Head and Neck Surgery, University of Toronto; ^4^ Department of Molecular Genetics, University of Toronto

**Keywords:** Neuroblastoma, propranolol, beta-adrenergic receptor, p53, p73

## Abstract

Neuroblastoma (NB) is a pediatric tumor of the sympathetic nervous system, which is often associated with elevated catecholamines. More than half of patients with metastatic NB relapse and survival is extremely poor with current therapies. In a high-throughput screen of FDA-approved drugs we identified anti-NB activity for the nonselective β-adrenergic receptor antagonist propranolol hydrochloride.

Propranolol inhibited growth of a panel of fifteen NB cell lines irrespective of *MYCN* status, and treatment induced apoptosis and decreased proliferation. Activity was dependent on inhibition of the β2, and not β1, adrenergic receptor, and treatment resulted in activation of p53 and p73 signaling in vitro. The majority of NB cell lines and primary tumors express β2 adrenergic receptor and higher mRNA levels correlate with improved patient survival, but expression levels did not correlate with in vitro sensitivity to propranolol. Furthermore, propranolol is synergistic with the topoisomerase I inhibitor SN-38 and propranolol inhibits growth of NB xenografts *in vivo* at doses similar to those used to treat infants with hemangiomas and hypertension. Taken together, our results suggest that propranolol has activity against NB and thus should be considered in combination treatments for patients with relapsed and refractory NB.

## INTRODUCTION

The sympathetic nervous system cancer neuroblastoma (NB) is the most common extra-cranial solid tumor in childhood. NB is characterized by a broad range of clinical behaviors and biological heterogeneity. Despite intensive treatments that include chemotherapy, radiation, surgery, stem cell transplant and immunotherapy, and an increased understanding of molecular characteristics of NB, more than 50% of children with metastatic NB relapse, and survival from recurrent NB is less than 10% [1,2]. Thus novel therapies are needed for patients with newly diagnosed and relapsed NB. Repurposing existing medications prescribed for non-malignant diseases has been successfully used to identify active agents for pre-clinical testing for cancers including NB. FDA-approved drugs have been identified to have anti-cancer properties by unbiased high-throughput cell based screening assays using drug libraries or studies of candidate drugs with proposed mechanisms of actions that may target tumor promoting signaling pathways [3-6]. In pediatrics a recent systematic review of repurposing drugs found that almost 10% of drugs approved for primary use in children have been repurposed for new indications for pediatric oncology or other pediatric indications [7]. In contrast to development of new compounds repurposing approved drugs may result in faster or more streamlined initiation of clinical trials. In order to identify novel therapies for relapsed NB we previously conducted high-throughput screens with the Prestwick library (Prestwick Chemical, Inc) of 1,120 FDA approved drugs using a panel of NB cell lines [6]. In addition to chemotherapies with known efficacy in NB, we identified additional compounds with anti-NB activity including propranolol hydrochloride, a nonselective beta-blocker that competitively inhibits the action of epinephrine (EPI) and norepinephrine (NE) on β1- and β2-adrenoreceptors (AR). Although originally used for the treatment of hypertension and other cardiovascular disorders, recently propranolol has been shown to be effective and safe for the treatment of large hemangiomas in infants [8].

Additional rationale supports potential anti-cancer, and specifically anti-NB effects for β-blockers. Catecholamines and their metabolites increase proliferation of several different cancer cell types *in vitro* [9,10] and patients with NB often have elevated serum and urinary catecholamines [11]. Anti-tumor activity of propranolol *in vitro* has also been demonstrated for many cancer cell lines including pancreatic, breast, gastric, head and neck squamous cell carcinoma and leukemia [12-17]. Furthermore, retrospective epidemiology studies suggest that cancer patients treated with beta-blockers have improved outcomes [18-21]. Based on these clinical findings, the pro-proliferative effects of catecholamines, and the safety profile for propranolol in children we hypothesized that the beta blocker propranolol may have potential efficacy in NB [22,23].

In this study we demonstrate that propranolol reduces the viability of human NB cell lines through the inhibition of proliferation and induction of apoptosis. The β2-AR is expressed on NB cell lines and primary tumor tissue, and higher levels of expression correlate with improved survival. However, the level of expression does not correlate with sensitivity to propranolol. Propranolol treatment in vitro is associated with induction of apoptosis and the pro-apoptotic p53 family proteins p53 and p73. Propranolol treatment at doses similar to those used to treat infants with hemangiomas also results in growth inhibition of NB xenografts and induction of p53 *in vivo*. Our findings suggest that propranolol, alone or in combination with chemotherapy, may be an effective agent in NB.

## RESULTS

### Propranolol inhibits NB growth, viability, and proliferation

**Figure 1:**
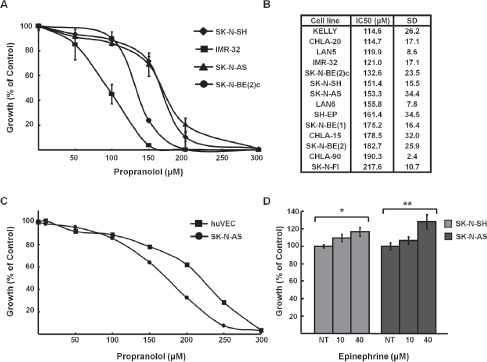
Propranolol inhibits neuroblastoma growth *A*, Growth curves of NB cell lines treated with indicated doses of propranolol for 72h. *B*, IC50 values for fifteen NB cell lines. IC50 measurement was performed with alamarBlue (triplicate). Results represent the average of three experiments expressed as a mean percentage of untreated control cells. *C*, HUVEC cells were treated with indicated concentrations of propranolol and IC50 was determined with alamar blue (triplicate). Results represent the average of three experiments expressed as a mean percentage of untreated control cells. *D*, SK-N-SH and SK-N-AS cells were stimulated with indicated doses of EPI for 24h. Cell growth was measured using alamarBlue (triplicate) and normalized to untreated controls. Data shown are representative of three independent experiments and are expressed as means of triplicates ± s.e. *p=0.04, **p=0.011

Propranolol is a non-selective β-blocker that competitively inhibits the action of EPI and NE on β1-and β2-AR. To determine the effect of propranolol on NB, a panel of fifteen established human NB cell lines representing a range of genetic profiles (eg. status of *MYCN* amplification, *p53* mutation, 1p and 11q LOH) were treated with increasing doses of propranolol to determine the half-maximal inhibitory concentration (IC50) using alamarBlue, an indicator of metabolic activity and cellular health (Figure 1A). The IC50s ranged from 114 μM to 218 μM (Figure 1B), doses similar to those reported for propranolol in non-NB cancer cell lines, which range from 100-300 µM [12-17]. The IC50 for human umbilical vein endothelial cells (HUVEC) was similar to the IC50 measured for the majority of NB cells (Figure 1C). In contrast to the growth inhibition observed in response to treatment with β-antagonists we detected an increase in proliferation of NB cells in response to β-agonist EPI, at levels consistent with those reported for other non-NB cell types [10,24] (Figure 1D).

**Figure 2:**
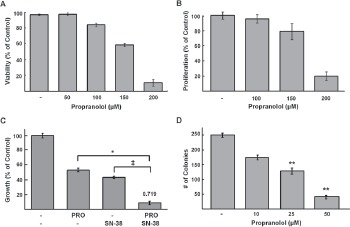
Propranolol inhibits NB viability and proliferation and is synergistic with SN-38 *A*, SK-N-AS cells were incubated with indicated doses of propranolol for 24h and cell viability was determined using trypan blue exclusion method. Results are expressed as a mean percentage of trypan blue negative cells (live cells); data shown are representative of three independent experiments and are expressed as means of triplicates ± s.d. *B*, SK-N-SH cells were treated with indicated doses of propranolol for 24h. Cell proliferation was assessed by BrdU incorporation. Results are a mean percentage of control cells. *C*, alamarBlue assays were performed on SK-N-AS cells treated with increasing doses of propranolol and SN-38 at a ratio of 10,000:1 (100μM propranolol: 0.01μM SN-38) for 48hr. Results are expressed as percentage compared to controls. The combination index (CI) was determined based on the Chou-Talalay method is shown on the top of the bar representing combination treatment * p= 0.008, ‡ p= 0.0009. *D*, Concentration-dependent effects of propranolol on clonogenic growth. SK-N-AS cells were treated for 14 days with propranolol at indicated doses or media alone (control); colonies were detected by staining with crystal violet. Bars represent the mean number of colonies of triplicate wells from three independent experiments ± s.d; ** p <0.001 students t-test.

We next asked whether growth inhibition in response to propranolol was due to changes in viability and/or proliferation. Decreased viability and proliferation were detected by trypan blue (Figure 2A) and BrdU incorporation assays (Figure 2B), respectively. To determine whether topoisomerase I inhibitors such as irinotecan, which is commonly used to treat NB relapse, result in enhanced efficacy cells were treated with the combination of propranolol and the irinotecan active metabolite SN-38. In comparison to cells treated with either drug alone a greater decrease in cell viability was detected for cells treated with the combination (Figure 2C). This effect was synergistic based on the combination index (CI) of 0.719 calculated by the Chou-Talalay method. Propranolol is rapidly metabolized and thus, usually delivered in at least two divided doses per day. For our *in vitro* assays a single dose was delivered prior to performing specific growth or proliferation assays. In order to determine longer-term effects of lower doses of treatment *in vitro* we used a focus formation assay that assesses self-renewal capacity, in which cells were treated for 14 days with propranolol replaced daily. There was a significant decrease in the number of foci or colonies in a dose dependent manner following 14 days of treatment (Figure 2D). Compared to control cells, foci were reduced by 50% following treatment with 25μM propranolol and 84% with 50μM propranolol.

**Figure 3:**
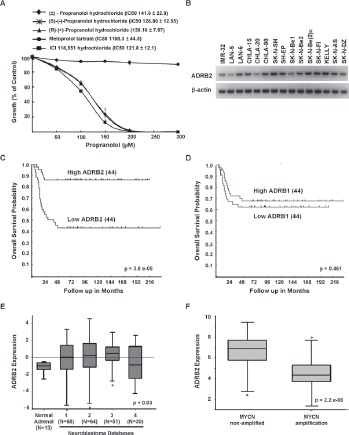
Growth inhibition is specific to β2-blockers and β2-AR is expressed in NB *A*, SK-N-SH was treated with increasing doses of either (±)-propranolol HCl enantiomers nd a β1-AR specific antagonist, metoprolol tartrate, or a β2-AR specific antagonist, ICI 118,551 HCl for 72 hours. Results re expressed as percentages of growth compared with untreated ontrols. Data shown are representative of three independent xperiments and are expressed as means of triplicates ± s.d. For ach β-AR antagonist, the IC50 of each cell line is shown with he corresponding legend. *B*, β2-AR (ADRB2) expression in NB cell lines as demonstrated by semi-quantitative PCR. *C-D*, Cumulative overall survival probability curves of patients with euroblastoma based on expression of ADRB2 mRNA [C] or ADRB1 mRNA [D] (N=88 patients, Versteeg dataset). The cut-ff for these analyses is the median expression level, high and ow represent levels of expression for 44 patients in each group elative to the mean. *E*, Relative ADRB2 mRNA expression in ormal adrenal tissue and neuroblastoma samples. *F*, Relative ADRB2 mRNA expression in MYCN non-amplified and mplified neuroblastoma samples. Kaplan-Meier curves and Box blots were generated from the R2: microarray analysis and isualization platform (http://r2.amc.nl), which includes data ubmitted by different investigators (Databases: 1, Versteeg; 2, Delattre; 3, Hiyama; 4, Lastowska).

### Growth inhibition is specific to β2-blockers and β2-AR are expressed in NB

To determine whether the growth inhibitory effects of propranolol were a result of its inhibitory effects on β1- or β2-AR, SK-N-BE(2)c cells were treated with (±)-propranolol HCl, the β1-specifc antagonist metoprolol tartrate or the β2-specifc antagonist ICI 118,551 hydrochloride. While the β1-specifc antagonist had no effect on viability, the β2-specifc antagonist ICI118,551 was slightly more potent than propranolol, suggesting that propranolol may induce cell death in NB via β2-AR specific antagonism (Figure 3A). We also tested three different enantiomers of propranolol HCl ((S)-(-)-propranolol HCl, (R)-(+)-propranolol HCl, (±)-propranolol HCl) and detected no significant difference in potency between the racemic mixture and the two enantiomers (Figure 3A). We next asked whether NB cells express β2-AR mRNA (ADRB2) and whether the levels observed vary in cell lines with differing sensitivity to propranolol. All NB cell lines tested expressed ADRB2 and there was no direct correlation between the IC50 and the level of ADRB2 mRNA detected (Figure 1 and Figure 3B). Most cultured NB cell lines are isolated from patients with metastatic NB. To determine whether the levels of ADRB2 in primary NB tumors correlate with prognosis or known biological risk factors we examined expression data in publically available databases [25-29]. Using data from the R2: microarray analysis and visualization platform (http://r2.amc.nl), which includes annotated data on 88 patients (Versteeg dataset) [29], a univariate analysis showed that higher levels of ADRB2 mRNA correlate with improved survival (Figure 3C). These findings were confirmed in a second cohort, Neuroblastoma Oncogenomics Database (http://pob.abcc.ncifcrf.gov/cgi-bin/JK) (data not shown). In contrast, mRNA levels of the β1-AR ADRB1 did not predict differences in prognosis (Figure 3D). Furthermore, in comparison to the expression of ADRB2 in normal adrenal tissue the relative expression of ADRB2 is higher in NB tumors (Figure 3E). ADRB2 is detected in tumors from patients with all stages of disease, but relatively higher levels were observed in patients without *MYCN* amplification (Figure 3E) and those <1 year of age (data not shown).

**Figure 4:**
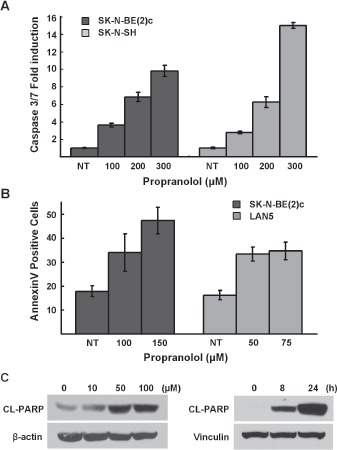
Propranolol induces apoptosis *A*, SK-N-SH and SK-N-BE(2)c were treated with indicated doses of propranolol for 5h and cellular caspase 3/7 activities were analyzed. Results are representative of three independent experiments, performed in triplicate and are expressed as mean fold-induction over the control cells ± s.d. *B*, LAN5 and SK-N-BE(2)c cells were treated with indicated doses (24 hours) and live cells were analyzed for induction of annexinV positivity by flow cytometry. Results are expressed as mean fold-induction over the untreated control ± s.d. *C*, SK-N-BE(2)c cells were treated with indicated doses for 48h and CHLA-20 cells were treated with 150 μM of propranolol for indicated durations and then analyzed for PARP cleavage by immunoblot analysis.

**Figure 5:**
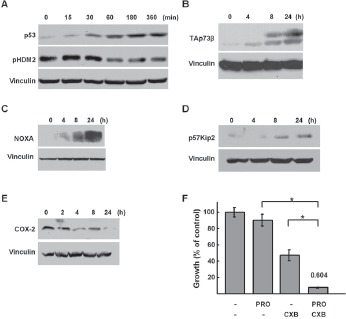
Regulation of p53 family signaling by propranolol *A*, p53 and pHDM2 western immunoblots of lysastes from SK-N-SH cells treated with propranolol (100 μM) for the indicated durations. *B-E*, TAp73β, NOXA and p57^kip2^, COX-2, western immunoblots of lysates from SK-N-AS cells treated with 150 μM of propranolol for the indicated durations. F, alamarBlue assays were performed on SK-N-AS cells treated with increasing doses of propranolol and Celecoxib (CXB) at a ratio of 2:1 (150μM propranolol: 75 μM CXB) for 48hr. Results are expressed as percentage compared to controls. The combination index (CI) was determined based on the Chou-Talalay method is shown on the top of the bar representing combination treatment * p<0.0001

### Propranolol induces apoptosis in neuroblastoma

To determine if the decreases in NB cell growth and viability following propranolol treatment was due to induction of cell death, we performed three assays to detect apoptosis. First, we detected an 8- to 15-fold increase in caspase 3 and 7 activity (Figure 4A). In response to propranolol treatment there was a 2.5 fold increase in the number of live NB cells undergoing apoptosis as detected by AnnexinV positivity (Figure 4B). Finally, the levels of poly-ADP-ribose polymerase (PARP) cleavage increased in both a dose- and time-dependent manner (Figure 4C).

### Propranolol increases p53 and TAp73 and pro-apoptotic target genes

We next addressed potential downstream apoptotic signaling mechanisms involved in propranolol induced NB cell death. A number of signaling pathways have been implicated in EPI-mediated activation of β2-AR, including activation of mitogen-activated protein kinase/ extracellular signal-related kinase (MAPK/ERK) and changes in p53. Hara and colleagues recently demonstrated that in response to catecholamines including EPI and NE, β2-AR signaling led to DNA-damage and activation of β-arrestin-1 as well as AKT, which phosphorylates HDM2 (pHDM2), resulting in p53 degradation [30]. Therefore, we predicted that inhibition of the β2-AR with propranolol may result in increased levels of p53, which is required for apoptosis in response to common chemotherapies as well as other drugs studied in preclinical NB models [31,32]. Following treatment with propranolol, we detected an increase in p53 as well as a time-dependent decrease in the levels of the phosphorylated form of the p53 negative regulator HDM2 (pHDM2) (Figure 5A). Some of the NB cell lines that are sensitive to propranolol express mutant and/or inactive p53 proteins (eg. SK-N-B(E)2, SK-N-AS, SK-N-FI). We and others have shown that the p53 paralogue p73 can activate p53 target genes that mediate apoptosis in response to a number of chemotherapeutic agents [33]. Thus, we asked whether the protein levels of the pro-apoptotic p73 isoform TAp73β changed in response to propranolol. Following propranolol treatment TAp73β isoform levels increased (Figure 5B). In addition, the protein levels of the p53 and TAp73 downstream pro-apoptotic target gene proteins NOXA and p57^kip2^ also increased (Figure 5C, 5D). We next examined whether activation of p53 and p73 might be due to DNA damaging effects of propranolol. As expected levels of γH2AX, a marker for double strand DNA breaks, increased in response to doxorubicin; however, no increase in γH2AX was detected following propranolol treatment (Supplemental Figure 1). We examined other downstream pathways previously implicated in the proliferative response to EPI in other cell types. Specifically, in response to propranolol treatment we observed decreases in levels of cyclo-oxygenase-2 (COX-2) (Figure 5E). Since we and others have demonstrated anti-NB activity for the COX-2 inhibitor celecoxib via effects of p53/p73 and VEGF signaling [31,34,35] the effects of celecoxib in combination with propranolol were assessed. Interestingly, celecoxib was synergistic with propranolol based on the combination index (CI) of 0.604 (Figure 5F).

**Figure 6:**
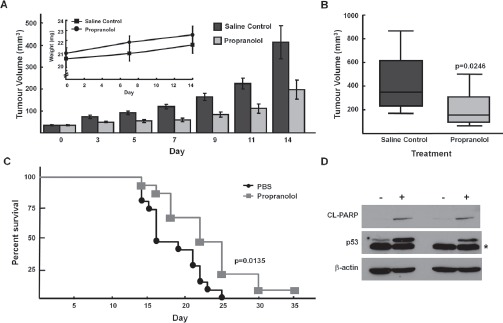
Propranolol inhibits neuroblastoma tumor growth *in vivo* NOD/SCID mice with SK-N-AS xenografts were treated with propranolol (1mg/kg bid) by subcutaneous injection for 14 days. *A*, Tumour volumes measured at indicated days post-injection of cells. Inset, absolute mean body weight, in grams, after the beginning of the treatment. Columns depict mean values; bars, SE. *B*, Box plot depicting mean tumour volumes of control (vehicle) treated and propranolol treated tumours at day 14. Each treatment group was composed of 10 mice (p<0.05). Bars depict mean values and error bars represent 95% confidence intervals. p values (2-tailed) were calculated using students t-test and are compared to control groups at indicated time points. *C*, Kaplan–Meier survival curve of NOD/SCID mice from control-treated (black, circle; N=15) or propranolol-treated (grey, square; N=15) tumours. Wilcoxon: p= 0.0135, Log-Rank: p=0.0062. *D*, p53 and cleaved-PARP western immunoblots of lysates from SK-N-AS xenografts harvested day 14 (lanes 1,2) and day 22 (lanes 3,4) post vehicle control or propranolol (1mg/kg/dose bid). Note: asterisk refers to a faster migrating non-specific band detected in p53 immunoblot of SK-N-AS xenograft lysates.

### Propranolol inhibits growth *in vivo*

The doses of propranolol required to inhibit NB growth in short term assays *in vitro* were higher than those predicted to be achievable *in vivo*. However, since focus formation assays demonstrated efficacy with lower doses of propranolol, we next investigated the effects of propranolol on growth of SK-N-AS NB xenografts. When tumors reached 50mm^3^, mice were injected subcutaneously with 2mg/kg/day (1mg/kg/day twice a day) for fourteen days. The tumors of mice treated with propranolol demonstrated slower growth as compared to PBS vehicle-treated control mice. The mean tumor volume of propranolol-treated group was lower than the control group on day 14: 197.6 ± 44.3 mm^3^ versus 414.5 ±76.6 mm^3^, unpaired t-test p = 0.0246) (Figure 6A, 6B). Notably, the mean body weight of propranolol-treated mice was not statistically significantly lower that that of the vehicle treated (control) mice, suggesting that propranolol exposure was not resulting in significant short-term toxicity (Figure 6A). Mice treated with propranolol also had a prolonged survival in comparison to control mice (Figure 6C). Importantly, similar to our *in vitro* results we also detected induction of p53 and cleaved PARP in lysates from xenografts of mice treated with propranolol (Figure 6D).

## DISCUSSION

In our study, we found that the non-selective β2-AR antagonist propranolol inhibits NB growth and that β2, but not β1, antagonists were active against a panel of human NB cell lines at doses comparable to those previously reported *in vitro* for other cancer cell types. Propranolol induced apoptosis that correlated with induction of p53 and the p53 paralogue TAp73 as well as activation of downstream p53/p73 target genes. In addition, propranolol was synergistic with the topoisomerase inhibitor SN-38 and the COX-2 inhibitor celecoxib, and had efficacy *in vivo* against NB xenografts.

Although initially used in the treatment of hypertension and arrhythmias in adults and children, recently propranolol has become standard therapy in infants with large hemangiomas, proliferative lesions of vascular endothelial cells [8,36]. β-ARs and their antagonists have also been shown to have efficacy in several pre-clinical cancer models. Catecholamines including EPI and NE as well as the β-AR agonist isoproterenol have pro-proliferative effects on cancer cell lines derived from lung, pancreatic, and oral squamous cell carcinomas [9,10,24,37-40]. β-ARs are expressed on multiple malignant cell types and treatment with propranolol has been reported to reduce viability, and in some cases induce apoptosis, of cancer cell lines including pancreatic, gastric, leukemia, melanoma, and oral squamous cell carcinoma [12-14,17,18,41]. Recent epidemiology studies provide further evidence for the potential anti-cancer effects of β-blockers. A ten year longitudinal study found that women with breast cancer receiving β-blocker therapy for hypertension had fewer metastases, recurrences and decreased mortality [16]. In a second retrospective study of breast cancer patients, women receiving the nonselective β-blocker propranolol, but not those receiving β1-specifc antagonists, were significantly less likely to present with advanced stage tumors [19]. Similar trends have been reported in retrospective analyses of patients with other types of tumors including melanoma [18,42,43].

Rationale for the use of β-AR antagonist propranolol specifically in NB include the observation that the majority of NB tumors produce elevated levels of catecholamines including EPI and NE, both of which have been shown to have pro-proliferative effects when added to cells in culture [9,10,24,37-40]. We also detected enhanced growth of NB cell lines in response to EPI as well as growth inhibition in response to propranolol treatment in 15 NB cell lines at concentrations similar to those reported in cell lines derived from other cancers. Although *MYCN* status has been linked to the sensitivity of NB cell lines to several drugs including aurora kinase inhibitors we did not detect a difference in the IC50 values based on *MYCN* amplification [44,45]. The cellular signaling events that mediate apoptosis in response to propranolol are poorly understood. Recent data demonstrated that in primary and transformed cells, β2-AR signaling activated by catecholamines leads to βarrestin-1 activation, which results in enhanced AKT-mediated phosphorylation and inactivation of HDM2. In response to β2-AR blockade with propranolol we detected decreased levels of the phosphorylated form of HDM2 (pHDM2) and increased p53 protein. We further found that treatment with propranolol resulted in induction of TAp73. The full-length form of the p53 paralogue p73, TAp73, can also mediate apoptotic responses to chemotherapies as well as other anti-cancer agents with efficacy in NB cells, including chemotherapies, aurora kinase inhibitors, and COX-2 inhibitors, particularly when p53 is inactivated [33,35]. Accordingly, we found induction of TAp73β and activation of p53/p73 pro-apoptotic genes NOXA and PUMA as well as p57^KIP2^, a cyclin-dependent kinase inhibitor, the promoter for which has previously been identified to contain p53 and p73 response elements [46-48]. Furthermore, recent reports support a role for p53 family proteins in the regulation of genes involved in angiogenesis, raising the possibility that previously reported anti-angiogenic effects of propranolol may be in part mediated by p53 or p73 [49]. The mechanism for induction of TAp73 in response to propranolol is not clear, but is not associated with DNA-damage as measured by γH2AX foci. Instead, the increased TAp73 but may be in part related to the decreased levels of COX-2 observed following propranolol treatment since COX inhibitors have been shown to induce TAp73β protein and activity [35].

β2-specifc AR antagonists, but not β1-specfc AR antagonists, inhibited the growth of NB cell lines *in vitro*; suggesting that expression of the β2-AR may be required for propranolol-induced cell death. Interestingly, the specific RNA levels of the β2-AR did not correlate with propranolol sensitivity *in vitro*, but were fairly similar in the 15 cell lines that we tested. β2-AR mRNA (ADRB2) is expressed in the majority of NB tumors, as well as cell lines derived from metastatic tumors, at levels that are higher than those detected in normal adrenal tissue. Interestingly, relative levels of ADRB2, but not ADRB1, correlate with favorable prognosis. The observation that high levels of ADBR2 are associated with more favorable prognosis is similar to findings for patients with other types of cancers, including oral squamous cell carcinoma (OSCC), leukemia, and prostate cancer [50-52]. Our findings demonstrate that the majority of patients have tumors that express β2-AR and thus, if expression is required for response, we would predict that most NB tumors would be sensitive to propranolol. However, it is also possible that growth inhibitory effects of propranolol may not be due specifically to inhibition of the β2-AR and may instead be due to off-target effects.

Although the dose required to induce apoptosis *in vitro* was high (100μM range), tumor inhibition in SK-N-AS xenografts was observed at doses similar to those used to treat infants with hemangiomas (1mg/kg/day bid). Interestingly, the IC50 for hemangioma-derived cells *in vitro* (100μM) is similar to those for NB cells *in vitro* [53]. Pharmacokinetic data suggests that in patients treated with propranolol the peak serum concentrations range from 200-400 ng/ml, which is equivalent to 0.77-1.5µM [54]. There are many possible explanations for this significant discrepancy between *in vitro* and *in vivo* effects. First, propranolol has been reported to inhibit pro-angiogenic factors, such as VEGF, and thus, *in vivo* tumor inhibition may be due to effects on endothelial cells in the blood vessels that surround the tumor. Although we did not detect significant difference in the IC50 for HUVEC endothelial cells in comparison to NB cells *in vitro*, which is supported by previous reports [55], a recent publication by Pasquier and colleagues demonstrates that propranolol can potentiate the anti-angiogenic effects vincristine *in vivo* , which included decreased capillary vessel formation [56]. Another potential mechanism may involve effects on the immune system. The β-adrenergic agonist metoproterenol inhibits Natural killer (NK) cell function, and its effects are reversible by treatment with propranolol [57]. Thus, future studies examining effects of propranolol on the tumor microenvironment or anti-tumor NK function may be warranted.

Despite the discrepancy between *in vitro* and *in vivo* concentrations the doses effective against NB SK-N-AS xenografts were similar to those used in regimens for hypertension and those reported for children with hemangiomas (2-3 mg/kg/day) [8,22,36,58]. The *in vitro* synergy with the irinotecan metabolite SN-38 and COX-2 inhibitors also supports potential combination therapy with topoisomerase inhibitors, such as irinotecan or topotecan, or COX inhibitors such as celecoxib in relapsed patients including those with central nervous system (CNS) metastases since propranolol, which is highly lipophilic, is known to cross the blood brain barrier and concentrate in the central nervous system (CNS) [59]. In a recent study by Pasquier and colleagues propranolol alone did not demonstrate single agent efficacy when administered to TH-*MYCN* transgenic mice, but was synergistic with vincristine and vinblastine, two agents commonly used for relapsed NB [60].

Taken together our results suggest that targeting the β2-AR with propranolol, which has been used in children for almost 50 years and more recently in infants with large hemangiomas, may be effective in children with NB. NB cell lines derived from metastases are sensitive to propranolol mediated growth inhibition. Although additional studies are required to determine whether β2-AR protein levels or other biomarkers predict which tumors are most sensitive to beta blockade *in vivo* our results suggest the majority of NB tumors express ADRB2 and thus may respond to β-AR antagonists. Therefore, together with recent retrospective studies that demonstrate improved outcomes for adult cancer patients receiving β-blockers, our data suggests that propranolol should be considered in combination with chemotherapies for the treatment of patients with relapsed or refractory neuroblastoma and possibly for neuroblastoma patients requiring anti-hypertensive therapy. Furthermore, early phase clinical trials for refractory neuroblastoma utilize tumor biopsies for expression profile analyses in an effort to identify FDA-approved drug combinations that may be effective for individual patients, and thus, our findings may provide additional rationale to include propranolol in regimens for subsets of these patients [61,62].

## MATERIALS AND METHODS

### Cell culture and Drugs

Established human neuroblastoma cell lines, KELLY, CHLA-20, LAN-5, IMR-32, SK-N-BE1, SK-N-BE(2), SK-N-BE(2)c, SK-N-SH, SK-N-AS, LAN-6, SH-EP, CHLA-15, CHLA-90, SK-N-FI were cultured in RPMI containing 10% fetal bovine serum (Invitrogen, Burlington, ON, Canada) and obtained from American Type Culture Collection and Dr. Patrick Reynolds (Children's Oncology Cell Bank). Cells were incubated at 37°C and 5% CO2 tissue culture incubator. Drugs: (±)-Propranolol hydrochloride, (S)(-)-propranolol HCl, (R)(+)-propranolol HCl, Metoprolol tartrate, ICI 118,551 hydrochloride and Epinephrine-HCL (Sigma, St. Louis, MO, USA) were dissolved in water; SN-38 (Tocris Bioscience, Bristol, UK) and Celecoxib (Toronto Research Chemicals, Toronto, ON, CA) were dissolved in DMSO.

### Apoptosis assays

Cells were seeded into a 96-well plate at 1.5x10^4^ cells per well and treated 24 hours later with propranolol for five hours. The combined caspase-3 and caspase-7 activity was measured using the Apo-ONE caspase 3/7 Assay Kit, according to manufacturer's instruction (Promega, Madison, WI, USA). Fluorescence was read using a luminometer (Spectra MAX Gemini EM, Molecular Devices) at 499/521 nM wavelengths. For annexinV assays cells treated with propranolol for 24 h were harvested and stained with propidium iodide (PI) and APC-AnnexinV (Dead Cell Apoptosis kit, Invitrogen) according to the manufacturer's instructions. The PI negative population was selected and analyzed for AnnexinV staining using a FACS Canto II flow cytometry (Beckton Dickinson).

### Cell Viability, Proliferation and Focus formation Assays

IC50 values were determined with alamarBlue assay (Invitrogen, Burlington, ON, Canada). 3-6 x10^3^ cells were plated in 96-well plates and treated with indicated doses of propranolol for 72 hours. At 48 hours, alamarBlue was added (10% of total volume) and then incubated overnight. The fluorescence was measured using a spectrophotometer at excitation 530 nm and emission 590 nm (Spectra MAX Gemini EM, Molecular Devices). Cell proliferation was assessed using Bromodeoxyuridine (BrdU) (Cell Signaling, Danvers, MA, USA). 4000 cells were treated with indicated drug and then incubated with BrdU (10μM) for 4h and detected using anti-BrdU antibody. The absorbance was measured at 450nM using a spectrophotometer plate reader (*VersaMax* tunable **microplate reader, Molecular Devices**). Trypan blue exclusion assay (Gibco, St. Louis, MO, USA) was used to measure viability following 24 hours of propranolol treatment. Live and dead cells (trypan blue positive) were counted in triplicate using a hemocytometer. For the colony or foci formation assay cells were seeded (10^2^/ well in six-well plates) in 2 ml of growth medium and incubated overnight at 37°C. Propranolol or media was added at the specified concentrations. Every 24h fresh propranolol and growth medium was added, and the plates were incubated at 37°C. Fourteen days after seeding, colonies were fixed in 70% ethanol and stained with 10% methylene blue. Colonies of 50 cells were counted.

### Western immunoblot analysis

Whole cell extracts were prepared using EBC buffer (50mM Tris (pH 8.0), 120 mM NaCl, 0.5% NP-40) with protease inhibitors (Roche Diagnostics, Laval, QC, Canada) and total protein concentration was determined using Bradford reagent (Bio-Rad laboratories, Hercules, CA, USA). Equal amounts were resolved by SDS-PAGE and transferred to nitrocellulose membrane. The membranes were blocked in 5% milk/tris-buffered saline with tween (TBST), probed with indicated primary antibodies, horseradish peroxidase-conjugated secondary antibodies (Thermo Fisher Scientific, Rockford, IL, USA). Protein was isolated from tumors by homogenization in RIPA buffer containing protease inhibitors. Proteins were detected by an enhanced chemiluminescene system (Super Signal West Pico, Thermo Fisher Scientific, Rockford, IL, USA). Primary antibodies include: Cl-PARP, pHDM2ser116 (Cell Signaling Technology, Beverly, MA, USA), TAp73/GC-15, p53/DO-1/AB-6 (Oncogene, La Jolla, CA, USA), Vinculin (Upstate, Lake Placid, NY, USA), NOXA/114C307 (Novus Biological LLC, Littleton, CO, USA), p57^kip2^ /c-20 (Santa Cruz Biotechnology, Santa Cruz, CA, USA).

### γH2AX immunostaining

SK-N-AS cells seeded in uncoated Labtek chamber slides (Nunc) (80,000 cells/chamber), were grown for 24 hrs and treated for an additional 24 hrs with 0.5mL media containing the indicated. Cells were fixed with 4% PFA, permeabilized with 0.2% Triton X-100 for 5 min and blocked with 6% Normal Goat Serum in 0.5% BSA for 1 hr. Slides were stained with primary antibody Phospho-Histone H2AX (Ser139) (20E3) Rabbit mAB (Cell Signaling) (1:400) overnight at 4°C, secondary Alexa Fluor 488 Goat Anti-Rabbit IgG (H+L) (Life Technologies) (1:5000) for 1hr at room temperature and DAPI (1:5000) and visualized using spinning disc confocal microscopy. 50 felds-of-view (FOV) were captured and cells were scored positive for the presence of γH2AX foci and compared to the number of foci in untreated cells.

### Semi-quantitative PCR

Total RNA was extracted using TRIzol (Invitrogen). cDNA was generated using the Omniscript RT Kit (Qiagen, Mississauga, ON, Canada) and amplifed by semi- using the Taq DNA polymerase Kit (Qiagen). RT-PCR conditions: initial denaturation (95°C/3min), 40 cycles of denaturation (94°C/1min), annealing (55°C/1min) and extension (72°C/1min), final extension (72°C/ 10min). Primer sequences for ADRB2 are: 5'-GAGCAAAGCCCTCAAGAC-3' and 5'-TGGAAGGCAATCCTGAATC-3', β-actin 5'-CTGGAACGGTGAAGGTGACA-3' and 5'-AAGGGACTTCCTGTAACAATGCA-3'. The RT-PCR products were resolved using a 1.5% agarose gel.

### Combination Index and Statistical analyses

For combination treatment, cells treated in a ratio of 10,000:1 (Propranolol:SN-38) or 2:1 (Propranolol:CXB) for 48 hours were subjected to alamarBlue assay. Using CalcuSyn Software (Biosoft, Cambridge, UK) the combination index (CI) was calculated [63,64]. CI<1 and CI>1 indicated synergism and antagonism, respectively, and CI=1 indicates an additive effect. Comparisons between 2 groups were done using unpaired Student t-test with the Graphpad Prism software (GraphPad Software, Inc., version 3.0). The Kaplan–Meier method was used to determine survival of mice. All p values < 0.05 were considered statistically significant.

### Xenograft studies

NOD/SCID (non-obese diabetic/severe combined immunodeficiency) mice were injected with 1.5x10^6^ SK-N-AS cells in PBS and Matrigel Basement Membrane Matrix (100μM) (BD biosciences, Franklin Lakes, NJ USA) subcutaneously in the left flank. When xenografts reached 50mm^3^ mice were treated with propranolol (Sigma, Israel) dissolved in phosphate buffered saline (PBS) or PBS alone (control) administrated subcutaneously at a dose of 1 mg/kg twice daily for up to 35 days. Tumor growth was monitored three times a week and tumor volume (mm^3^) was calculated. Mice were sacrificed when tumors were greater 500mm^3^. Tumor volumes were compared on Day 14 in two independent experiments (N=5 and N=10 per group). Survival curves were generated by combining two experiments (N=15 per group).

## Supplemental Figure


