# Supplementary material for: Anti-tumor activity of the beta-adrenergic receptor antagonist propranolol in neuroblastoma
Source: Oncotarget. 2013 Nov 4;5(1):161–72. doi: 10.18632/oncotarget.1083 (PMC3960198; doi:10.18632/oncotarget.1083)
Supplement: Supplementary file 1 [file oncotarget-05-0161-s001.pdf]

Anti-tumor activity of the beta-adrenergic receptor antagonist propranolol in neuroblastoma - Wolter etal

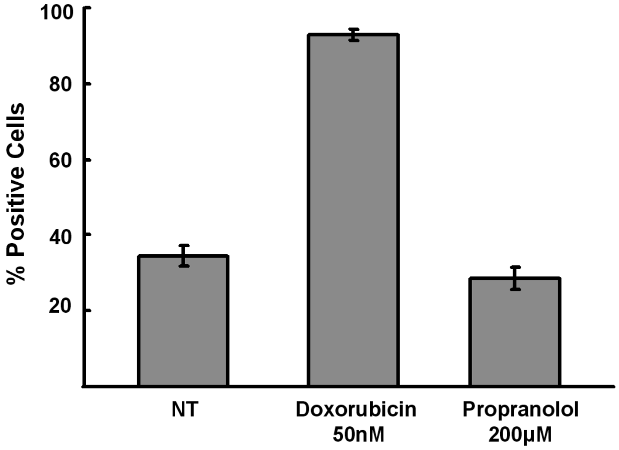

Supplemental Figure 1: Immunostaining of γH2AX positive cells following treatment with 24h 50nM doxorubicin or 200µM propranolol. Quantification of γH2AX-phosphorylated positive cells pooled from 50 randomly selected fields-of-views (FOV) from are shown.
